# Bilateral basal ganglia ischemia associated with COVID-19: a case report and review of the literature

**DOI:** 10.1186/s13256-021-03165-x

**Published:** 2021-11-22

**Authors:** Khurram Khaliq Bhinder, Ahsun Rizwan Siddiqi, Muhammad Junaid Tahir, Hamza Maqsood, Irfan Ullah, Zohaib Yousaf

**Affiliations:** 1 Shifa International Hospital, Islamabad, Pakistan; 2 POF Hospital, Wah Cantt, Rawalpindi, Pakistan; 3 Lahore General Hospital, Lahore, 54000 Pakistan; 4 Nishtar Medical University and Hospital, Multan, Pakistan; 5 Kabir Medical College, Gandhara University, Peshawar, Pakistan; 6 Hamad Medical Corporation, Doha, Qatar

**Keywords:** SARS-CoV-2, Infection, Brain, Neurology, Radiology, Neuroimaging

## Abstract

**Background:**

Coronavirus disease 2019, caused by the severe acute respiratory syndrome coronavirus 2, has a broad clinical spectrum, from asymptomatic to multi-organ dysfunction. Acute cerebrovascular events associated with coronavirus disease 2019 are mainly due to the severe acute respiratory syndrome coronavirus 2-induced prothrombotic state. Bilateral basal ganglia ischemia is rarely reported.

**Case presentation:**

We report the case of a 64-year-old Asian (Pakistani) gentleman who presented initially with fever, cough, and shortness of breath, likely due to respiratory involvement by severe acute respiratory syndrome coronavirus 2. Later, he developed bilateral lower limb pain, followed by confusion and decreased level of consciousness. Accentuated large hypodense opacities were seen in the left and right basal ganglia, with mass effects on the left frontal horn.

**Conclusion:**

This case demonstrates the importance of neuroimaging in the effective management of patients with neurological signs associated with coronavirus disease 2019.

## Background

Coronavirus disease 2019 (COVID-19) results in a broad spectrum of clinical manifestations [1]. A third of the infections are symptomatic. The predominant symptoms are consistent with respiratory involvement and include fever, cough, dyspnea, and fatigue. However, a severe illness characterized by acute respiratory failure, septic shock, and multi-organ failure leading to death is also reported [1]. In addition, patients with COVID-19 are at an increased risk of developing prothrombotic complications, including deep venous thrombosis, acute limb ischemia, and ischemic stroke [2]. Studies estimate that 0.8–6.4% of coronavirus patients experience an ischemic infarct [3]. However, bilateral basal ganglia ischemia is rarely reported in COVID-19. We present a middle-aged gentleman with COVID-19 who developed bilateral basal ganglia ischemia. Such cases highlight the atypical neurologic sequelae of COVID-19, likely secondary to the associated prothrombotic state.

## Case presentation

A 64-year-old Asian (Pakistani) diabetic and hypertensive gentleman initially presented with fever, cough, and progressive shortness of breath for 3 days. Prior to this presentation, the patient was on a combination of metformin–sitagliptin 1000 mg/50 mg twice daily per oral (PO), atorvastatin 40 mg once daily, and valsartan–hydrochlorothiazide 160 mg/25 mg daily PO. Patient was a never smoker and not an alcohol user. Patient was married and had two healthy kids. He was an accountant by profession. There was no personal or family history of malignancy, venous thromboembolism (VTE), or bleeding or clotting disorder. On presentation to the hospital, his blood pressure was 110/70 mmHg, pulse rate was 100 bpm, respiratory rate was 23/min, and oxygen saturation was 89% on room air. On examination, the patient had coarse crackles in bilateral lower lung fields, which did not change character with cough. Neurological examination was negative for meningeal signs and the Glasgow Coma Scale (GCS) was 15/15. The rest of the physical examination was unremarkable. A chest X-ray (CXR) demonstrated bilateral diffuse heterogeneous infiltrates, more pronounced on the right side. Due to high clinical suspicion, a nasopharyngeal swab was collected for SARS-CoV-2 Reverse transcription Polymerase Chain Reaction (RT PCR), which was positive, confirming COVID-19.

A high-resolution computed tomography (HRCT) of the chest showed multifocal, multilobar ground-glass opacities in central and peripheral locations, more pronounced in middle and lower lobes, with septal thickening, giving a classical crazy paving appearance. Radiological Society of North America (RSNA) guidelines were used to calculate disease burden based on HRCT, and the score was 21/40 (severe disease burden) [4]. During the hospital stay, the patient was admitted to the COVID-19 isolation unit, where he received moxifloxacin, piperacillin/tazobactam, enoxaparin, and dexamethasone, based on the local COVID-19 treatment protocol at that time.

On day three of his hospital stay, the patient developed bilateral lower limb pain without any erythema, change in color, or change in the size of his lower limbs. An arterial and venous Doppler ultrasound was negative for occlusion or deep venous thrombosis. On day four, the patient was noted to have developed confusion and decreased level of consciousness. The GCS dropped from 15/15 to 10/15. The patient was emergently sedated, intubated, and mechanically ventilated to protect the airway.

**Fig. 1:**
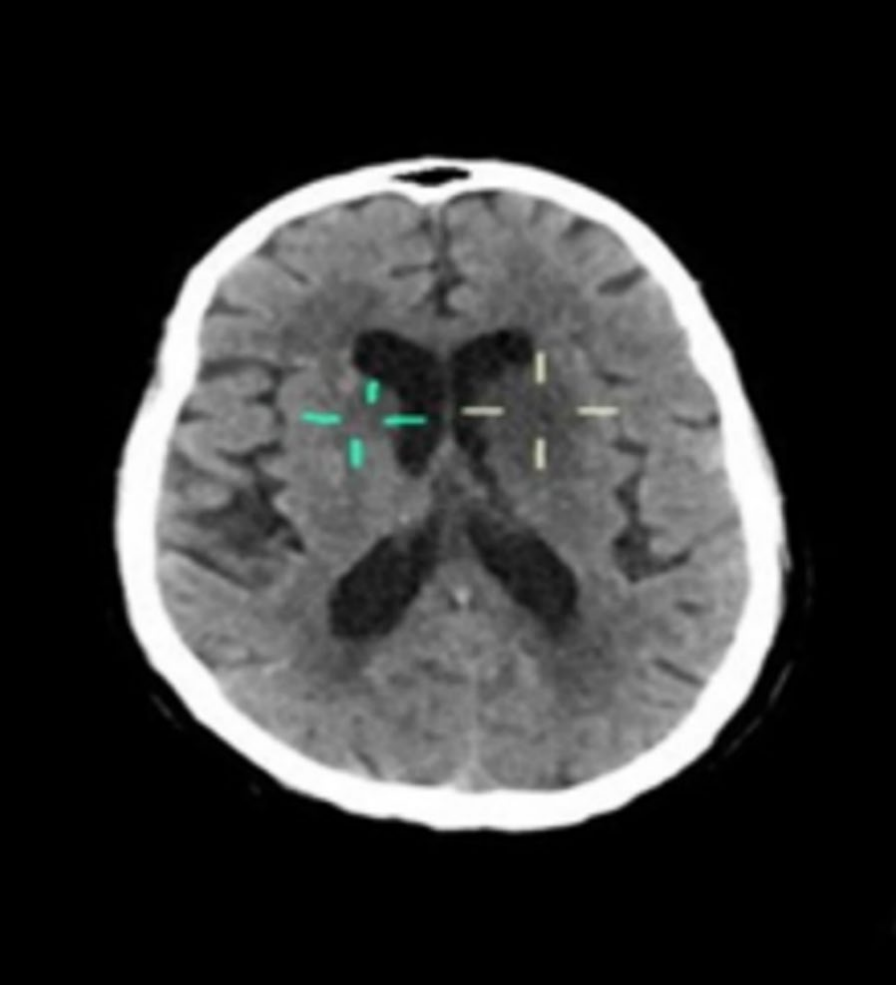
Non-contrast computed tomography (NCCT) of the brain showing accentuated hypodensities in left basal ganglia (involving caudate nucleus, lentiform nucleus, and anterior limb of internal capsule) and a smaller hypodensity in the right caudate nucleus

An urgent non-contrast computed tomography (NCCT) of the brain (Fig. 1) revealed accentuated hypodensities in the left basal ganglia (involving caudate nucleus, lentiform nucleus, and anterior limb of internal capsule), with mass effect on the ipsilateral frontal horn. Another smaller hypodensity in the right caudate nucleus was also seen. With a diagnosis of ischemic stroke, the patient received aspirin and continued to receive statin and antihypertensives. However, thrombolysis was not considered as the exact time of onset of symptoms was not documented, and the patient had well-developed ischemic changes on the NCCT, signifying being past the thrombolytic period.

Over the next few days, the patient received care in the intensive care unit, but his general condition deteriorated, with increasing oxygen requirements. On day 27 of the hospital stay, the patient developed cardiac arrest. Cardiopulmonary resuscitation was unsuccessful, and he passed away. Autopsy was declined by the family and, hence, could not be done.

## Discussion

Our case describes a gentleman with severe COVID-19 pneumonia-associated bilateral basal ganglia hemorrhage. The incidence of bilateral basal ganglia ischemia during coronavirus infection is rare. A literature search performed on PubMed on 07 June 2021 using the Boolean operator strategy of “(basal ganglia) AND (ischemia) AND (COVID-19)” showed four results. The screening of abstracts revealed two cases by Matos *et al.* and Kulick-Soper *et al.* reporting basal ganglia ischemia [15, 16]. Matos *et al.* attributed the stroke to vasculopathy and Kulick-Soper *et al.* attributed the ischemia to possible hypoxic-ischemic injury. The clinical and radiological features of both studies are listed in Table 1. The common feature is presence of altered mental status. However, unlike other cases, paresthesia, apathy, or movement abnormalities were not present.

**Table 1:** Clinical features of ischemia of basal ganglia reported in the literature

Author	Patient’s age	Initial presentation	Neurological manifestations	CT findings
Matos *et al*.	42 years	Fever, dry cough, myalgia, asthenia, and hyposmia	Altered mental status, slowness of movements, and apathy	Multiple hypodense lesions involving the white matter, basal ganglia, and thalamus
Kulick-Soper *et al*.	52 years	Bilateral hand paresthesia followed by fever, cough, dyspnea, headache, and confusion	Bilateral hand paresthesia and confusion	Symmetric hypoattenuation in the bilateral globus pallidus with surrounding small foci of hyperattenuation

The increased risk of ischemic cerebrovascular complications associated with COVID-19 is an emerging concern. As the COVID-19 pandemic has progressed, there has been a wide array of COVID-19-related neurologic manifestations, including encephalopathy, encephalitis, stroke, and Guillain–Barre syndrome [5]. COVID-19 has been linked to the hypercoagulable and prothrombotic state implicated in ischemic stroke [6]. The pathophysiology of COVID-19 helps explain the neurovascular effects of the disease. SARS-CoV-2, upon entry into the human body, binds the angiotensin-converting enzyme 2 (ACE-2) receptor, which is expressed on pulmonary type 2 pneumocytes, cardiac myocytes, and vascular endothelial cells [7]. Vascular endothelial cells are critical to the regulation of vascular permeability, maintaining hemostasis, and regulating hemolysis [8]. This explains their role in COVID-19 vasculopathy and thrombosis. In particular, SARS-CoV-2 releases interleukin-1 (IL-1), interferon-gamma, and tumor necrosis factor (TNF)-alpha, which not only leads to increased vascular permeability and endothelial injury, but also to increased platelet activation factor and inhibition of thrombomodulin, leading to thrombosis, as suggested by Shams *et al*. [9]. In patients with COVID-19, native arterial occlusions are observed in both younger and older individuals, and more often in males than females in the general population [10].

The basal ganglia and thalamus are paired deep gray matter structures affected by various conditions, including systemic or metabolic diseases, degenerative vascular disorders, and infections [11]. Basal ganglia infarction is a type of cerebral infarct with unique clinical manifestations. Old age, diabetes, and hypertension are considered risk factors for cerebral ischemic lesions [12]. In an observational study on COVID-19 patients with neurological symptoms, about 5.5% of the patients were found to have basal ganglia lesions [13]. COVID-19 is postulated to have neuroinvasive potential. In addition, the raised inflammatory markers may indicate a hyperinflammatory state, which may play a role in cerebral ischemia [14].

Our study has certain limitations. First, we were unable to investigate which underlying diseases significantly increase the relative risk of presenting with neuroinflammation and cerebrovascular disorders. Second, due to clinical circumstances, we were unable to obtain the magnetic resonance imaging for the patient. Further studies are required to fully elucidate the association between COVID-19-associated basal ganglia ischemia and the underlying pathophysiology. This case further signifies the importance of neuroimaging in the effective management of patients who present with an altered mental status and neurological signs during their COVID-19 illness.

## Conclusion

COVID-19-associated basal ganglia ischemia may be multifactorial secondary to the prothrombotic state, hypoxic-ischemic injury, or vasculitis. Therefore, understanding the pathophysiology of basal ganglia involvement in COVID-19 and utilizing neuroimaging tools is vital in managing the neurological sequelae of SARS-CoV-2.

## Data Availability

Not applicable.

## Abbreviations

COVID-19: Coronavirus disease 2019
SARS-CoV-2: Severe acute respiratory syndrome coronavirus 2
RT-PCR: Reverse transcription-polymerase chain reaction
HRCT: High-resolution computed tomography
RSNA: Radiological Society of North America
NCCT: Non-contrast computed tomography
